# Do fetuses move their lips to the sound that they hear? An observational feasibility study on auditory stimulation in the womb

**DOI:** 10.1186/s40814-016-0053-3

**Published:** 2016-03-01

**Authors:** Nadja Reissland, Brian Francis, Louisa Buttanshaw, Joe M. Austen, Vincent Reid

**Affiliations:** 1 Department of Psychology, Durham University, Durham, UK; 2 Department of Mathematics and Statistics, Lancaster University, Lancaster, UK; 3 Department of Psychology, Lancaster University, Lancaster, UK

**Keywords:** Prenatal stimulation, Fetal hearing, Fetal mouth movement

## Abstract

**Background:**

We investigate in this feasibility study whether specific lip movements increase prenatally when hearing a particular sound. We hypothesised that fetuses would produce more mouth movements resembling those required to make the sound stimulus they heard (i.e. mouth stretch) compared with a no-sound control group who heard no specific auditory stimuli. Secondly, we predicted that fetuses hearing the sound would produce a similar number of mouth movements unrelated to the sound heard (i.e. lip pucker) as the no-sound group of fetuses.

**Methods:**

In an observational feasibility study, 17 fetuses were scanned twice at 32 and 36 weeks of gestation, and two different types of mouth movements were recorded. Three fetuses received an auditory stimulus, and 14 did not. A generalised mixed effects log-linear model was used to determine statistical significance.

**Results:**

Fetuses in the sound group performed one specific mouth movement (mouth stretch) significantly more frequently than fetuses in the no-sound group. A significant interaction between group and gestational age indicates that there was differential change in this specific movement as age increases (*X* ^2^ = 7.58 on 1 df, *p* = 0.006), with the no-sound group showing a decline of 76 % between 32 weeks and 36 weeks (*p* < 0.001), whereas the sound group showed no significant change over time (*p* = 0.41). There was no significant difference between the sound group and no-sound group in the frequency of lip puckering—the second, unrelated mouth movement (*p* = 0.35).

**Conclusions:**

These results suggest that a sound stimulus is associated with an increase in specific, rather than general, mouth movements. The results are informative for the development of infant speech and potentially could also lead to a diagnostic test for deafness in utero. More research is needed to replicate this research with a randomised design and with a range of different auditory stimuli which would be produced with different mouth movements, such as “o” which would be seen as pursed lips.

**Electronic supplementary material:**

The online version of this article (doi:10.1186/s40814-016-0053-3) contains supplementary material, which is available to authorized users.

## Background

From birth, infants produce silent movements resembling the lip movements necessary for speech. A seminal study reported by Trevarthen [1] in which one 7-week-old girl silently produced lip and mouth movements which resembled the mouth movements of a female speaker reading a word list has been interpreted as the basis of the intention to speak. These mouth movements indicated that very young infants when hearing language in their first weeks after birth produce mouth and lip movements similar to those necessary to replicate speech. In addition, infants just after birth have been shown to imitate silently mouth movements required to produce language sounds, even when no sound was produced [2].

One way in which this ability could develop prenatally was proposed by Green and Wilson [3]. They argued that randomly produced lip and jaw movements during fetal development could conceivably create sensorimotor pathways that could serve as precursors of early speech. They based their conjecture on the well-established suggestion that neuronal firing resulting in leg and hand movements is a precursor of walking and grasping movements (e.g. [4, 5]). Neuronal activation elicits early limb movements and these limb movements in turn help to consolidate the pathways which shape purposeful movements such as reaching or walking (e.g. [4, 5]). Hence, early activation of specific mouth and jaw movements such as a jaw drop with elongation of lips in the vertical axis could be a precursor necessary for speech sounds such as “a” and pursing lips could be a precursor to producing the lip movement necessary for the sound “o”.

Prenatal cognitive development has been tested in relation to sound and light stimulation. For example, Horimoto et al. [6] reported that fetuses between 32 and 34 weeks of gestation showed a high incidence of mouth movements that were later during gestation correlated with non-rapid eye movements. However, the mouth movements which have been reported in previous studies are general movements that do not have specific definitions, such as a smile or grimace.

The following sequential order of development has been found for specific fetal oral movements, namely jaw opening, jaw closing, tongue movement and lip movement [7], whereby spontaneous movements of the jaw appear as early as 11 weeks during prenatal development [8]. Regarding first vocalisations in infancy, jaw opening and closing are primary movements during babbling [9] which can be distinguished from other mouth openings such as smiling [10]. Green et al. [11] investigated the sequential development of jaw and lip control and found in an analysis of children aged 1 to 6 years of age that it developed sequentially, with jaw movements preceding lip movements.

By the second trimester, the fetal auditory cortex is reorganised by external stimulation (e.g. [12, 13]). In the study by Kisilevsky et al. [13], which examined maturation of fetal responding to airborne auditory stimuli, differential responding occurred as a result of fetal maturation during the third trimester. Hence, we selected two gestational ages in the third trimester to examine whether we might find maturational changes in reaction to an auditory stimulus. If fetuses were exposed to auditory rhythmic stimulation [14], then this might be reflected in their production of movement patterns, specifically movement patterns of the jaw and lips. Given that fetal mouth movements develop but have to date not been analysed to the level of specificity required, it is essential to establish whether there is a relationship between mouth movements and sound stimulation. If jaw and lip movements are produced prenatally in response to sound before the ability to produce speech develops, then this would support the argument that precursors of language are rooted in fetal development. In particular, we argue that if types of jaw and lip movements vary between fetuses who do not hear any sounds during scans and fetuses who hear specific sounds, there might be reason to believe that lip and jaw movements are precursors of silent pre-speech movements which can be observed in neonates.

The production of the auditory stimulus presented in this study involved predominantly jaw movements, allowing us to determine whether the response to this stimulus was specific (i.e. only jaw movements were produced) or general (i.e. jaw and lip movements were produced). In order to investigate this question, we studied two groups of fetuses: one that was presented with the auditory stimulus during scanning and a second no-sound control group of fetuses that was not presented with any sound stimulus. We expected that those fetuses presented with the specific sound would produce mouth movements consistent with the sound when contrasted with another mouth movement that typically manifests with the same frequency in normally developing fetuses. This feasibility study was designed to establish whether it is possible to test fetal fine-grained reactions to specific sound stimulation.

## Methods

### Ethics

Ethical permission for the feasibility study was obtained from the County Durham and Tees Valley 2 Research Ethics Committee (REC Ref: 08/H0908/31 and County Durham and Tees Valley 2 Research Ethics Committee REC Ref: 11/NE/03/61) and the research and development department of James Cook University Hospital, as well as the Durham University (Department of Psychology ethics committee). All mothers gave informed written consent.

### Stimulus

The reporting of this study follows the STROBE checklist (see Additional file 1). The auditory stimulus consisted of multiple presentations of the sound MA (/ma:/in the International Phonetic Alphabet). The MA sound was spoken by a female voice, was 0.40 s in length and was repeated 8 times with 0.80 s of silence between each presentation. This cluster of eight MA sounds was then repeated for the duration of the scan, with 6.0 s of silence between each cluster. The auditory stimulus was played on a Sandisk Sansa Clip portable MP3 player, attached to a Kitsound Boombar portable speaker. Following recommendations by Kruger et al. [15], who report research showing that the speaker should not be placed on the mother’s abdomen, for this study, the speaker was held at a distance of 3 cm above the mother’s abdomen near the ear of the fetus for the duration of the stimulus presentation. The sound pressure level at a distance of 3 cm from the speaker was 94 dB, measured with a Precision Gold N05CC Digital Sound Meter (with a measurement range of 30–100 dB with an accuracy of ±1.5 dB), although uterine attenuation will have reduced the sound level for the fetus by approximately 20–35 dB [16, 17]. The auditory stimulus contained frequencies between 0 Hz and 11 kHz, with most output in the 0.6–1.6-kHz and 2.6–3.6-kHz regions. These frequencies are audible to fetuses from 29 weeks of gestational age [16]. Although, in a study of newborn auditory matching, Chen et al. [18] only measured responding to an auditory stimulus during its presentation, we measured responding during the presentation of sound and during the intervening seconds of silence. The inclusion of short periods of silence following the presentation of sound was deemed appropriate as fetuses may be slower to respond to sound stimuli than newborns due to their relative immaturity. The short periods of silence allow the fetuses time to respond to the recently presented auditory stimuli. The relative frequencies of jaw and lip movements during the presentation of the auditory stimuli were analysed and compared with the no-sound control group who did not experience the auditory stimulus.

### Participants

Mothers who had completed their normal 20-week anomaly scans were invited to participate in this study. All fetuses participating in this study were completely healthy as determined by their 20-week scan. A convenience sample of 17 mothers was recruited for this feasibility study; three mothers whose fetuses were exposed to the auditory stimulus (1 boy and 2 girls), the sound group, and 14 mothers whose fetuses were not exposed to any stimulus (7 boys and 7 girls), the no-sound group. The no-sound group participants were recruited through the midwives of the antenatal unit of the James Cook University Hospital, Middlesbrough, UK, and sound group participants through the hypnobirthing group in London at the Harley Street Ultrasound Centre (*www.thewisehippo.com*) following approved ethical procedures. During consent and before each procedure, mothers were made aware that the scans were for research purposes and were not routine medical scans. Given that maternal stress, attachment, anxiety and depression are known to affect fetal behaviour (e.g. [19–22]), the two groups were assessed for these factors with the Perceived Stress Scale (PSS [23]), Antenatal Maternal Attachment Scale (AMAS [24]) and the Hospital Anxiety and Depression Scale (HADS [25]).

### Procedure

All participating mothers received scans at 32 and 36 weeks of gestational age, with fetuses being scanned for approximately 900–1200 s, with the maximum time of the scan determined by the British Medical Ultrasound Society (BMUS) guidelines. The scan times of 32 and 36 weeks were chosen as Kisilevsky et al. [13] identified that it was more likely to get reactions to sound in the later stages of pregnancy. The scans took place either in the Ultrasound Department of James Cook University Hospital, where mothers had previously undergone their routine 12- and 20-week medical scans or in the London Ultrasound Centre. The scanning took place with mothers lying in a darkened room on their back or on their side, depending on the position of the fetus and how comfortable mothers were. The fetal face and upper torso were visualised both by means of 4D colour full frontal or facial profile ultrasound recordings, as well as sequences of traditional 2D monochrome images. The scans were recorded for offline analysis with a GE Voluson E8 Expert Ultrasound System using a GE RAB4–8L Macro 4D Convex Array Transducer. Mothers were provided with a DVD copy of their scans.

### Measures

Scan recordings were used to code mouth movements using the Fetal Observable Movement System (FOMS) [26], an adaptation of the Facial Action Coding System [27], which has been found to be reliable in previous research [28, 29]. Following established procedures [29], two types of mouth movements were identified for analysis: *mouth stretch* and *lip pucker*.

Mouth stretch is defined by the lower jaw being pulled down by the action of the external pterygoids and digastric muscles, so that the mouth is actively opened. The opening is stretched such that the longest axis is the vertical plane. The cheeks are stretched and flattened and the skin on the chin also may become bulged.

Lip pucker, in contrast, is defined by the lips narrowing and pursing with the lips protruding forwards. This is caused by the incisivii labii superioris and incisivii labii inferioris muscles, which pull the corners of the lips medially. The lips usually appear as if contracted and the mouth opening will look smaller and rounded. There also may be some bulging of the chin as the skin of the chin is pulled upwards towards the lips. In normally developing fetuses, these two mouth movements are produced with relatively equal frequencies at the gestational ages scanned in this study [26].

The aim in this feasibility study was to analyse up to 600 s of codable scan for each fetal scan. Codable sections of the scan for the control scans were sections where the fetal face was visible and where the pocket of amniotic fluid was present to allow a clear image. For the sound group, coding occurred during the presentation of sound and during the intervening seconds of silence. As fetal movements differ as a function of the movement state of the fetus [30], it is essential that all of the fetuses were in an active state during scanning. Assessment of the movement behaviour showed that all fetuses were in the active states of 2F or 3F during scanning, as assessed by their gross body movements and eye movements according to Nijhuis et al.’s four-state categorisation [30], and not in states 1F (totally passive) or 4F (overly active).

### Statistical analysis

Reflecting the longitudinal structure of the measurements and the non-normality of the count responses, a Poisson log-linear mixed effects analysis [31] was used to assess the effect of experimental group and gestational age and the interaction between them using the *glmer* function of the *lme4* library in R [32]. A mixed effects analysis has been shown to be superior to standard repeated measures analysis of variance for experimental data [33]. Moreover, imbalance in the number of participants in each treatment arm and in the number of scans contributed by each mother can easily be accounted for. The analysis models the number of *mouth movements* of different types as a count variable adjusted by the length of analysed scan as an exposure variable and a random individual fetus effect. The individual random fetus effect allows for individual variability between fetuses in their overall propensity to mouth movements and is assumed to be normally distributed.

Formally, we can write the model as

$$ {M}_{it}\sim \mathrm{Poisson}\ \left({\lambda}_{it}\right) $$

with

$$ \begin{array}{c} \log \left({\lambda}_{it}\right)= \log \left(\mathrm{scan}\kern0.5em {\mathrm{length}}_{it}\right)+{\beta}_0+{\beta}_1\kern0.32em \mathrm{gestational}\kern0.5em {\mathrm{age}}_{it}+{\beta}_2\;{\mathrm{treatment}}_i\\ {}+{\beta}_3\;{\left(\mathrm{gestational}\kern0.5em \mathrm{age}\times \mathrm{treatment}\right)}_{it}+{u}_i\\ {}{u}_i\sim \mathrm{Normal}\left(0,{\sigma}_f^2\right),\end{array} $$

where *M*_*it*_ is the mouth movement count for fetus *i* at gestational age *t*, *λ*_*it*_ is the underlying Poisson rate, *β*_0_ to *β*_3_ are unknown regression coefficients and *σ*_*f*_^2^ is the individual within-fetus variance. The indices to the individual covariates show which of them vary over time in our model. Thus, gestational age is recorded at each scan, whereas the treatment condition (sound/no sound) is constant for each fetus.

A test for overdispersion for count data was carried out on the full interaction model using the methodology of Mancuso [34]. If the overdispersion test indicated no overdispersion, then significance of terms was assessed through analysis of deviance likelihood ratio test, examining changes of deviance between fitted models, and comparing to a chi-squared distribution with the appropriate number of degrees of freedom. Bolker et al. [35] provides full details of fitting and testing Poisson log-linear mixed effects for count data.

## Results

Comparing mothers in the two groups, we analysed their results on a number of scales. On the Perceived Stress Scale (PSS [23]), mothers in the sound group were similar to those in the no-sound group at 32 weeks (*t*(15) = 0.16, *p* = 0.87) and at 36 weeks (*t*(15) = 0.18, *p* = 0.86). Scores on the Antenatal Maternal Attachment Scale (AMAS [24]) also did not differ between mothers in the sound and no-sound control groups either at 32 weeks (*t*(15) = 0.85, *p* = 0.41) or 36 weeks (*t*(15) = 0.50, *p* = 0.63). The Hospital Anxiety and Depression Scale (HADS [25]) was used to obtain measures of both anxiety and depression. In terms of anxiety, mothers did not differ between the two groups at 32 weeks (*t*(15) = 0.41, *p* = 0.69) or 36 weeks (*t*(15) = 0.73, *p* = 0.48). Levels of depression were also similar between mothers in the two groups at both 32 weeks (*t*(15) = 0.13, *p* = 0.90) and 36 weeks (*t*(15) = 0.50, *p* = 0.62). In addition, mothers did not differ in age between the two groups (sound *M* = 30, no sound *M* = 29), *t*(15) = 0.32, *p* = 0.75.

Although we aimed to analyse 600 s of codable scans, not all scans produced 600 s of codable material. The mean amount of time that was analysed from the no-sound control group scans was 570 s at 32 weeks (SD = 97, range = 236–600) and 600 s at 36 weeks (SD = 0, range = 600–600). For the sound group, the mean scan length was 188 s at 32 weeks (SD = 121, range = 48–261) and 194 s at 36 weeks (SD = 42, range = 150–234). It should be noted that in order to account for the differences in scanning time, the average number of movements observed for each group at each gestational age was divided by the average scanning time for that group at that age. This number was then multiplied by 100 to give a relative frequency of movements per hundred seconds of codable scan time. Thus, frequency of movements can be readily compared between groups with differing scan times in descriptive Tables 1 and 2. Below, we report the results for “mouth stretch” and “lip pucker”.

**Table 1:** Average number of mouth stretches per scan and rates of mouth stretching per 100 s of scan [with 95 % confidence intervals] for the sound and control groups by gestational age

	Average number of movements observed per scan		Relative frequency (movements per 100 s of scan)	
	32 weeks	36 weeks	32 weeks	36 weeks
Sound	1.67 [0.63, 3.65]	2.67 [1.26, 5.23]	0.89 [0.33, 1.94]	1.38 [0.65, 2.60]
Control	2.50 [1.77, 3.44]	0.64 [0.32, 1.17]	0.44 [0.31, 0.60]	0.11 [0.15, 0.20]

95 % confidence intervals calculated using Byar’s method for rates [39]

**Table 2:** Average number of lip puckers per scan and rates of lip puckering per 100 s of scan [with 95 % confidence intervals] for the sound and control groups by gestational age

	Average number of movements observed per scan		Relative frequency (movements per 100 s of scan)	
	32 weeks	36 weeks	32 weeks	36 weeks
Sound	0 [0.00, 0.82]	0.33 [0.03, 1.55]	0 [0.00, 0.43]	0.17 [0.02, 0.80]
Control	2.14 [1.47, 3.01]	2.21 [1.53, 3.10]	0.38 [0.26, 0.53]	0.37 [0.26, 0.52]

95 % confidence intervals calculated using Byar’s method for rates [39]

### Mouth stretches

**Fig. 1:**
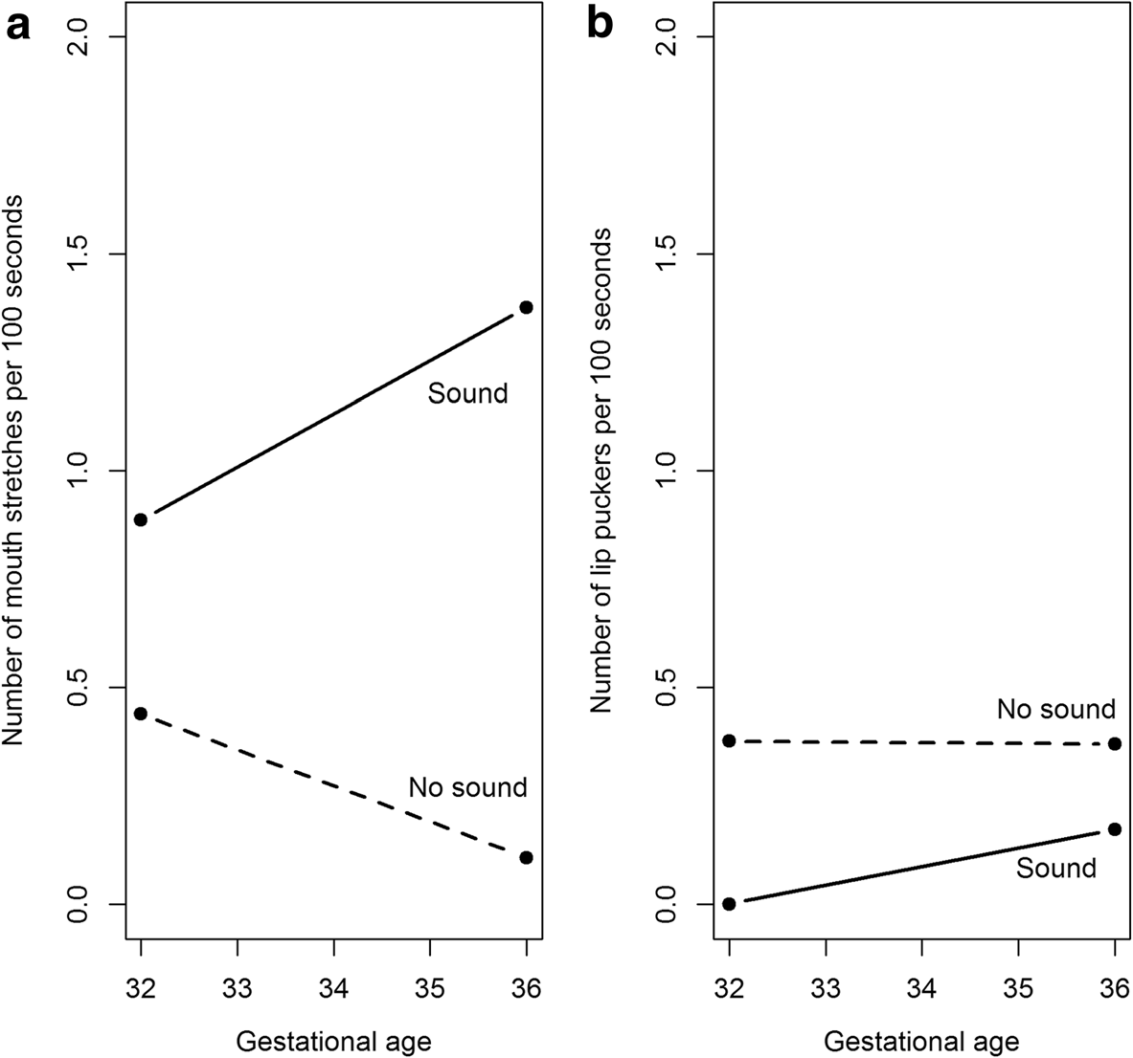
The effect of gestational age and presentation of an auditory stimulus on the frequency of (**a**) mouth stretches and (**b**) lip puckers

The mouth stretch mean counts from the fetuses at 32 and 36 weeks of gestational age can be seen in Table 1 and Fig. 1a. These suggest that fetuses at both gestational ages produce more frequent mouth stretches in the sound group compared with the no-sound control group. These observations were confirmed by the main effects Poisson linear mixed model. The overdispersion test gave a dispersion parameter estimate of 0.89, which is under one, and therefore indicates no overdispersion. There was a significant main effect of group, *X* ^2^ = 5.78 on 1 df, *p* = 0.01, and of gestational age (*X* ^2^ = 10.78 on 1 df, *p* = 0.001), demonstrating that fetuses in the sound group performed mouth stretches more frequently than fetuses in the no-sound control group, and there was a general trend towards fewer mouth movements as gestational age increased. When an interaction model was fitted, the interaction between group and gestation was also significant (*X* ^2^ = 7.58 on 1 df, *p* = 0.006), indicating that there was differential change in mouth stretch rate as gestational age increases. For the sound group, there was no evidence of a change in the rate of mouth stretch (*β* = 0.49, 95 % CI = [−0.71, 1.69], exp(*β*) = 1.63 , *p* = 0.41); for the no-sound group, there was a decrease of 76 % in the rate of mouth stretch (*β* = − 1.41, 95 % CI = [−2.17, − 0.65], exp(*β*) = 0.24, *p* < 0.001).

These data indicate that fetuses presented with the auditory stimulus MA produce more mouth stretches than fetuses presented with any specific auditory stimulus. However, it is possible that the presentation of the auditory stimulus triggered an increase in the number of mouth movements in general. In order to test this hypothesis, the frequency with which fetuses performed the second mouth movement, a lip pucker, was established. The lip pucker is a valid mouth movement for comparison as fetuses at 32 and 36 weeks of age not exposed to any specific stimulation show similar frequencies of mouth stretches and lip puckers. To corroborate this, we examined frequencies of lip pucker and mouth stretch in the control group. A paired-sample *t* test indicated that, pooled over the two gestational ages, there was no significant difference between the frequency of mouth stretches (*M* = 0.163 per minute) and lip puckers (*M* = 0.218 per minute), *t*(13) = 0.63, *p* = 0.54.

### Lip pucker

The lip pucker mean counts from the fetuses at 32 and 36 weeks of gestational age can be seen in Table 2 and Fig. 1b, showing only small differences in the frequencies with which fetuses pucker their lips depending on gestational age and group. Across both gestational ages, it appears that a similar amount of lip puckers were produced by the control group and the sound group. This was tested using the mixed effects Poisson model as before. Again, the overdispersion test indicated that no overdispersion was present in the data, with the dispersion parameter of 0.516 being less than one. Results indicate no statistically significant main effect of group, *X* ^2^ = 0.87 on 1 df, *p* = 0.35, no significant main effect of gestational age, *X* ^2^ = 0.01 on 1 df, *p* = 0.93, and no interaction between group and gestation, *X* ^2^ = 1.64 on 1 df, *p* = 0.20.

These data suggest that the frequency with which fetuses show lip pucker lip movements are independent of the presentation of an auditory stimulus. Additionally, there is no evidence that gestational age affects the rate of lip puckers. From this result, we can infer that the presentation of the auditory stimulus MA does not cause an increase in the frequency of mouth movements in general. Rather, it increases the frequency of a specific mouth movement corresponding to the MA sound, with a jaw drop and the mouth stretching in the vertical plane.

## Discussion

Results of this study indicate that fetuses respond to a specific sound MA with a specific mouth movement which mimics the sound heard, namely a mouth stretch which involves a jaw drop. There are a number of researchers who argue that given the precocity of the functional development of the auditory system, the abilities shown in newborn babies must have their origin in prenatal life [13, 36]. Given that research [37] has established that the fundamental frequency (F0) of vowels are well transmitted to the fetus, using the sound MA seemed to be an ideal candidate in this study. However, in order to eliminate the possibility that fetuses would respond with more mouth movements in general, we analysed the occurrence of another type of mouth movement, namely pursing of lips, which in the control no-sound group occurred with similar frequencies to mouth stretching. We found that upon hearing MA, fetuses did not respond with an increase in pursing of their lips. Given the results by Green et al. [11] who found that control over vertical movements of lips and jaw during speech developed sequentially with jaw movements preceding lip movements, this might explain why we found a relationship between hearing the MA sound and producing the mouth stretch. Ferronato et al. [38] suggest that postnatal speech stimuli are special stimuli which elicit specific behavioural reactions. They argue that the pairing of certain acoustic stimuli with defined motor activities (e.g. rhythmic sounds with rhythmic movements) demonstrate that the “human brain is primed with the body” (p. 3). This according to Ferronato et al. [38] could indicate that auditory input and behavioural output might play a role in the integration between external and internal information which is essential for learning in general and language acquisition in particular. In terms of differential responding to sounds over the duration of the third trimester, fetuses did not show a significant increase in the production of mouth movements as a consequence of maturation. However, there is a numerical increase in mean responses as gestational age increases, which needs to be further investigated.

Turning to the observed changes over time, we identified a significant interaction between the sound and no-sound groups in the slope of the rate of mouth stretch in response to the stimulus of “MA”. The no-sound group showed declining rate of mouth stretch, whereas the sound group showed no such decline. Fetal movements in general tend to decrease with gestational age. Our results provide evidence that appropriate mouth movements associated with specific stimuli do not decrease in this way.

This feasibility study was not a randomised control trial but used a convenience sample. However, we consider it unlikely that fetuses of mothers who did not consent to participate were different to those who participated in our study, and so there was minimal selection bias.

## Conclusions

This feasibility study established that it is possible to test specific fetal fine-grained reactions to sound stimulation. More research is needed to develop this feasibility study. Firstly, a randomised controlled trial is needed with balanced numbers of treatment and control participants. Additionally, a range of different auditory stimuli should be examined to determine whether the fetus is reacting to a specific MA sound or to any general auditory stimulus such as white noise. Additionally, a greater range of specific sounds could also be examined in order to fully explore how the fetus produces not only random mouth movements but specific pre-speech movements.

The potential implications of this work are twofold. Firstly, this work is likely to provide knowledge on the relationship of prenatal reaction to specific sounds and postnatal language development. Secondly, there is a possibility that lack of reaction of the fetus to specific sounds could be used as a prenatal diagnostic test for deafness.

## Additional file

Additional file 1:
**STROBE statement.** Checklist of items that should be included in reports of observational studies. (DOC 85 kb)
